# An Editor scientists dream of

**DOI:** 10.1101/gad.350500.123

**Published:** 2023-01-01

**Authors:** Nancy Hopkins

**Affiliations:** Koch Institute for Integrative Cancer Research, Massachusetts Institute of Technology, Cambridge, Massachusetts 02139, USA

Terri Grodzicker is the journal Editor that scientists dream of—brilliant and curious, fair and unbiased, highly critical, broadly and deeply knowledgeable, and not distracted by fads or hype. She is able to keep her professional distance while supporting hopeful authors, many of whom have become her lifelong friends. Terri was already a highly accomplished scientist when she became the Editor who would imagine and shape *Genes & Development*, making it into a premier journal. She's an Editor who has remained a scientist.

I met Terri at the Cold Spring Harbor Laboratory in 1970, when we were assigned as roommates at a Molecular Biology of Bacteria and Phage meeting. She was a lac operon person, and I was a phage λ researcher. A few years later, we both switched to tumor viruses. The molecular biology of phage and bacteria had made it possible to tackle cancer at the molecular level. Terri brought her training in molecular genetics to adenoviruses and adenovirus/SV40 hybrid viruses, making transformative contributions in collaboration with her colleagues Bob Tjian, Ray Gesteland, Phil Sharp, and Joe Sambrook.

Working with the above colleagues, Terri devised the first restriction fragment length polymorphism (RFLP) analysis of crossover events and mapping mutations of viral genomes, used adenovirus/SV40 hybrid viruses to make the first identification of nonsense mutations in a eukaryotic virus or eukaryotic organism (contemporaneously with studies in Capecchi's lab), and did research that led to the development of adenovirus vectors to overproduce viral or cellular proteins. While still a lab scientist at CSHL, Terri also began to participate in organizing the CSHL summer courses and meetings.

Given her research accomplishments and contributions to running the summer program, Terri might well have stayed on at the lab as a senior scientist for the remainder of her career or left for a faculty position in a university. Molecular biologists studying cancer and, for the first time in history, even women, were suddenly hot commodities on the job market.

One day, Terri told me she was considering a radical shift. Following the discovery of cancer genes, a number of tumor virologists were shifting their initial research goals. Instead of a shift, Terri was thinking of a leap to become the Editor of *G&D*. One obstacle, she told me, was that some scientists at CSHL didn't think a woman could be tough enough for the job. Today, such a view might seem inconceivable, but at the time it was a valid concern. Fortunately Jim Watson, an astute judge of people and at that time an early supporter of women scientists, overrode the objections and chose Terri to edit the journal.

That Terri's career choice would benefit me had not crossed my mind when she made such a bold change, but it did—particularly when I changed my own research directions twice. At midcareer, I switched from working on RNA tumor viruses to using zebrafish to identify genes essential for early development and disease. Intrigued by the potential of zebrafish, Terri asked Janet Rossant and me to write a review article for *G&D* explaining the advantages of the fish and why a number of senior scientists were shifting to work on it (Rossant and Hopkins 1992). By recruiting such a renowned developmental biologist as Rossant, Terri helped bring zebrafish to the attention of the molecular biology community, advanced the fish field, and incidentally gave me some welcome credibility in my new field and later a natural home for papers on insertional fish mutants (Allende et al. 1996; Amsterdam et al. 1999; Sun and Hopkins 2001). When our fish work, in collaboration with my MIT colleague Professor Jackie Lees, led my lab back to the cancer field, *G&D* was a perfect place to publish work on fish mutants in novel genes involved in checkpoints and replication (Sansam et al. 2006, 2010). Adding to the fun of publishing in *G&D* was the ease of the process, thanks to the superb staff Terri worked with at the journal.

Similarly, and particularly forward-seeing, Terri encouraged me to submit a review to *G&D* on a topic that had become a late-career passion and that was at the time far afield for many molecular biologists working in cancer; namely, prevention and early detection of cancer (Golemis et al. 2018). The review was coauthored by leaders in the fields of cancer prevention and drug development as well as molecular biology, with the hope that more molecular biologists would turn their skills to these approaches that have proven so powerful in reducing cancer deaths.

Looking back, one can see why Terri would prove to be such a brilliant and successful journal Editor. Having been a superb scientist herself, possessing a vast and ever-current knowledge gained from organizing and attending cutting-edge scientific meetings, and having genuine affection and empathy for scientists, she knew how and when to encourage us and when to rescue us from ourselves when we were wrongheaded.
